# Effects of ultrasound therapy on the synovial fluid proteome in a rabbit surgery-induced model of knee osteoarthritis

**DOI:** 10.1186/s12938-019-0637-2

**Published:** 2019-02-22

**Authors:** Qinglu Luo, Shuangquan Ji, Zhimi Li, Tao Huang, Siqin Fan, Qin Xi

**Affiliations:** 1 The Fifth Affiliated Hospital of Guangzhou Medicine University, No. 621, GangWan Road, HuangPu District, Guangzhou, 510700 Guangdong Province China; 2 College of Rehabilitation Medicine, Fujian University of Traditional Chinese Medicine, Fuzhou, 350122 Fujian China

**Keywords:** Ultrasound, Proteome, Synovial membrane, Osteoarthritis, Synovial fluid

## Abstract

**Background:**

Ultrasound (US) therapy may improve osteoarthritis symptoms. We investigated the effects of US on the synovial fluid (SF) proteome in a rabbit knee osteoarthritis (KOA) model to explore its therapeutic mechanisms.

**Methods:**

Sixteen healthy 6-month-old New Zealand white rabbits (eight male, eight female), weighing 2.5–3.0 kg, were randomly divided into groups A and B with eight rabbits per group. Both groups were subjected to right anterior cruciate ligament transaction. Six weeks after surgery, we treated the operated knee joint of group A rabbits with US and of group B rabbits with sham US for 2 weeks. The proteomes of knee joint SF from groups A and B rabbits were then analyzed using a label-free mass spectrometry (MS) quantification method.

**Results:**

We identified 19 protein sequences annotated by 361 Gene Ontology (GO) items. According to the Kyoto Encyclopedia of Genes and Genomes (KEGG) database of rabbit protein sequences, we then annotated the KO numbers of homologous/similar proteins to 32 relevant KEGG pathways. We extracted 10 significantly differentially expressed proteins among the 32 relevant KEGG messages/metabolism pathways. The proteins whose levels were decreased were apolipoprotein A-I (AopA-1), transferrin (TF), carboxypeptidase B2 (CBP2), arylesterase/paraoxonase (PON), fibrinogen alpha chain, and alpha-2-macroglobulin (A2M). The proteins whose levels were increased were molecular chaperone HtpG/heat shock proteins (htpG, HSP90A), decorin (DCN), pyruvate kinase (PK, pyk), and fatty acid-binding protein 4/adipocyte (FABP4, aP2).

**Conclusions:**

US therapy can alter protein levels in SF, which can decrease AopA-1, TF, CBP2, PON, fibrinogen alpha chain and A2M protein levels, and increase HtpG/HSP90A, DCN, PK/PKY, and FABP4/aP2 protein levels in SF of KOA, suggesting that the therapeutic mechanisms of US therapy on KOA may occur through changes in the SF proteome.

## Background

Osteoarthritis (OA) is a degenerative joint disease and a common cause of musculoskeletal disability that is often characterized by progressive destruction of articular cartilage and bone changes [1]. In addition to cartilage damage, subchondral bone, synovium and synovial joint structure also show pathological changes [2]. Vina and Kwoh [3] found that sociodemographic characteristics, obesity, genetic predisposition, high bone density/mass diet-related factors, specific bone/joint shapes, thigh flexor muscle weakness, joint malalignment, participation in certain occupational/sports activities, and joint injury were risk factors for OA. OA patients frequently require surgical treatment and 4 million American adults underwent total knee arthroplasty, accounting for 4.2% of the population aged 50 or more. More than half of the adults diagnosed with knee OA (KOA) in the United States receive total knee replacement [4]. However, the high cost of surgery, the limited life of biological materials implanted during surgery, the reduced tolerance of elderly patients to surgery and their susceptibility to infection, postoperative rehabilitation expenses and other factors are reasons to find more economical and effective ways to treat KOA [5]. The main pathological change of OA is progressive articular cartilage degeneration; however, synovitis is another major factor in the development of OA [6, 7]. Wang et al. [8] found that synovitis was related to cartilage defects and osteophytes. Histopathological examination showed the synovial membrane to have a serious and severe inflammatory response in OA [9].

Some researchers regard synovitis as the main cause of pain and edema in OA patients [10]. In OA the synovial volume increases because the synovial membrane becomes inflamed and secretes synovial fluid (SF) containing inflammatory molecules, such as interleukins (ILs) and tumor necrosis factor (TNF) [11]. Decreasing the degree of synovitis is an important aim in OA therapy.

Currently, many physical agents [such as ultrasound (US), pulsed electromagnetic field, ultra-shortwave] are used to treat the OA [11, 12]. However, US is superior to other physical agents, which can decrease the apoptosis of chondrocytes and delay cartilage degeneration [11]. However, the effect of US on the synovium and SF have not been documented. Some of the proteins in SF are secreted by the synovial membrane or chondrocytes or result from a diffusion process from abnormally elevated plasma levels. Therefore most studies have assayed blood markers as indicators of SF inflammation. But some inflammation markers of SF cannot diffuse into blood; therefore, direct investigation of SF should confirm the pathological change of OA.

Recently, studies of human or animal articular SF and cartilage proteomes by isobaric tags for relative and absolute quantification (iTRAQ) or mass spectrometry (MS) have been documented [13–15]. But whether US can affect the SF proteome has not been reported. We, therefore, studied the effect of US therapy on the SF proteome in a rabbit knee osteoarthritis (KOA) model.

## Materials and methods

### Animals

Sixteen healthy 6-month-old New Zealand white (NZW) rabbits (eight male, eight female), weighing 2.5–3.0 kg, were recruited. All protocols were in line with national legislation and guidelines for laboratory animal care and use of the People’s Republic of China Ministry of health, and were approved by the local ethics committee of Fifth Affiliated Hospital of Guangzhou Medicine University. All rabbits were killed by air embolization after the OA modeling operation and US intervention. NZW rabbits were provided by the Fujian University of Traditional Chinese Medicine animal testing center, batch number: SCXK (Shanghai) 2012-0011.

### Experimental instruments and software

The following apparatus were used. Easy-nLC Liquid Chromatograph (Thermo Scientific), Q Exactive Mass Spectrometer (Thermo Scientific), AKTA Purifier 100 (GE Healthcare), Multiscan FC Microplate Photometer (Thermo Scientific), Centrifuges (Eppendorf 5430R), Concentrator plus/Vacufuge (Eppendorf Concentrator Plus) Electrophoresis apparatus (GE Healthcare EPS601), Vertical Electrophoresis Tanks (SE260; GE-Healthcare), Thermofinnigan Easy-nLC 1000, Trap column, ASY column SC001 traps (RP-C_18_), Analysis column, EASY column SC200 (RP-C_18_), Maxquant (version 1.3.0.5), Perseus (version 1.3.0.4), MP Fastprep-24 Homogenate instrument (MP Biomedicals), Ultrasonic Cell Disrupter System, Constant temperature incubator, Vortex oscillator, ProteomeDiscoverer 1.4 (Thermo Scientific), MASCOT 2.2 (Matrix Science), Perseus 1.3 (M&M), 10 kDa Ultrafiltration centrifuge tubes, C18 Cartridge, Multiple Affinity Removal LC Column—Human 14/Mouse 3, iTRAQ Reagent‐4/8plex Multiplex Kit, Dissolution buffer (AB SCIEX), SCX chromatographic column, Polysulfoethyl (PolyLCInc, Maryland, U.S.A.).

### Animal grouping

Animals were randomly divided into group A (US therapy) and group B (sham US). Each group included eight rabbits.

### KOA model

Animals were subjected to right knee anterior cruciate ligament transection (ACLT) as previously described [16]. Rabbits were anesthetized by intraperitoneal injection of 5% chloral hydrate (3 ml/kg). The rabbit was then fixed in the animal-fixing frame, and the right knee shaved and sterilized. A section of the medial joint was revealed. We dislocated the patella and separated and cut the anterior cruciate ligament (ACL). ACLT was ensured by Lachman testing by the surgeon, a certified veterinarian. After washing the joint with sterile saline, the incision was sealed and sterilized. After the operation the rabbits were housed in separate cages, allowed to move freely, and were routinely cared for.

### Intervention

Six weeks after OA modeling, rabbits in group A were given the following US treatment: continuous mode, 1.5 W/cm^2^, 3 MHz, 10 min per session and five times per week, for 2 weeks. The animals in group B were treated by sham US (moving the US head as for group A animals, but without turning on the machine).

### Label-free quantitative proteomic analysis

#### Specimen collection

Fourteen days after intervention, the rabbits were anesthetized with 5% chloral hydrate. SF samples (0.5 ml) were taken from the knee joint cavity of the suprapatellar bursa, collected in an Eppendorf tube, and incubated for 5 min at room temperature. The solution was centrifuged by vacuum centrifugal concentration at 12,000×*g* for 5 min at room temperature and the supernatant was transferred to a new tube and stored at − 80 °C for future use.

### Process of measurement

#### Protein cleavage and quantification for a label-free experiment

SDT buffer was added to the sample. The lysate was sonicated and then boiled for 15 min. After centrifuged at 14,000*g* for 40 min, the supernatant was quantified with a BCA Protein Assay Kit (Bio-Rad, USA). The sample was stored at − 80 °C.

#### SDS-PAGE separation

Twenty micrograms of protein from each sample were mixed with 5× loading buffer and boiled for 5 min, centrifuged at 14,000×*g* for 10 min and separated by 12.5% SDS-PAGE (constant current 14 mA, 90 min). The protein bands were observed by Coomassie Blue R-250 staining.

#### Filter-aided sample preparation (FASP Digestion)

For each sample, 200 μg of proteins were mixed with 30 μl SDT buffer (4% SDS, 100 mM DTT, 150 mM Tris–HCl pH 8.0). The detergent, DTT, and other low-molecular-weight components were removed using UA buffer (8 M Urea, 150 mM Tris–HCl pH 8.0) by repeated ultrafiltration (Microcon units, 10 kD). Then 100 μl iodoacetamide (100 mM iodoacetamide in UA buffer) was added to block reduced cysteine residues and the samples were incubated for 30 min in the dark. The filters were washed with 100 μl UA buffer three times and then with 100 μl 25 mM NH_4_HCO_3_ buffer twice. Then the protein suspensions were digested with 4 μg trypsin (Promega) in 40 μl 25 mM NH_4_HCO_3_ buffer overnight at 37 °C, and finally the peptides were extracted. The peptides of each sample were desalted on C18 Cartridges [Empore™ SPE Cartridges C18 (standard density), bed I.D. 7 mm, volume 3 ml, Sigma], concentrated by vacuum centrifugation, and reconstituted in 40 µl 0.1% (v/v) formic acid. The peptide content was estimated by UV light spectral density at 280 nm using an extinction coefficient of 1.1 for a 0.1% (g/l) solution that was calculated on the basis of the frequency of tryptophan and tyrosine in vertebrate proteins.

#### Products of protein glycolysis analyzed by LCMS/MS

LC–MS/MS analysis was performed on a Q Exactive mass spectrometer (Thermo Scientific) that was coupled to Easy nLC (ProxeonBiosystems, now Thermo Fisher Scientific) for 60 min. The mass spectrometer was operated in positive ion mode. MS data was acquired using a data-dependent top 10 method dynamically choosing the most abundant precursor ions from the survey scan (300–1800 m/z) for HCD fragmentation. Automatic gain control (AGC) target was set to 3e6, and maximum injection time to 10 ms. Dynamic exclusion duration was 40.0 s. Survey scans were acquired at a resolution of 70,000 at m/z 200, and resolution for HCD spectra was set to 17,500 at m/z 200 and isolation width was 2 m/z. Normalized collision energy was 30 eV, and the underfill ratio, which specifies the minimum percentage of the target value likely to be reached at maximum fill time was defined as 0.1%. The instrument was run with peptide recognition mode enabled.

#### Maxquant software data analysis

We analyzed the data using MaxQuant software version 1.3.0.5 (Max Planck Institute of Biochemistry, Martinsried, Germany) [17]. The ProteinPilot parameters are presented in Table 1.

**Table 1:** Maxquant software parameters

Item	Value
Enzyme	Trypsin
Max missed cleavages	2
Max missed cleavages	2
Main search	6 ppm
First search	20 ppm
MS/MS tolerance	20 ppm
Fixed modifications	Carbamidomethyl (C)
Variable modifications	Oxidation (M), acetyl (protein N-term)
Database	See the project report
Database pattern	Reverse
Peptide FDR	≤ 0.01
Protein FDR	≤ 0.01
Time window (match between runs)	2 min
Protein quantification	Razor and unique peptides were used for protein quantification
LFQ [8]	True
LFQ min. ratio count	1

#### Statistics and bioinformatic analysis by Perseus software

**Fig. 1:**
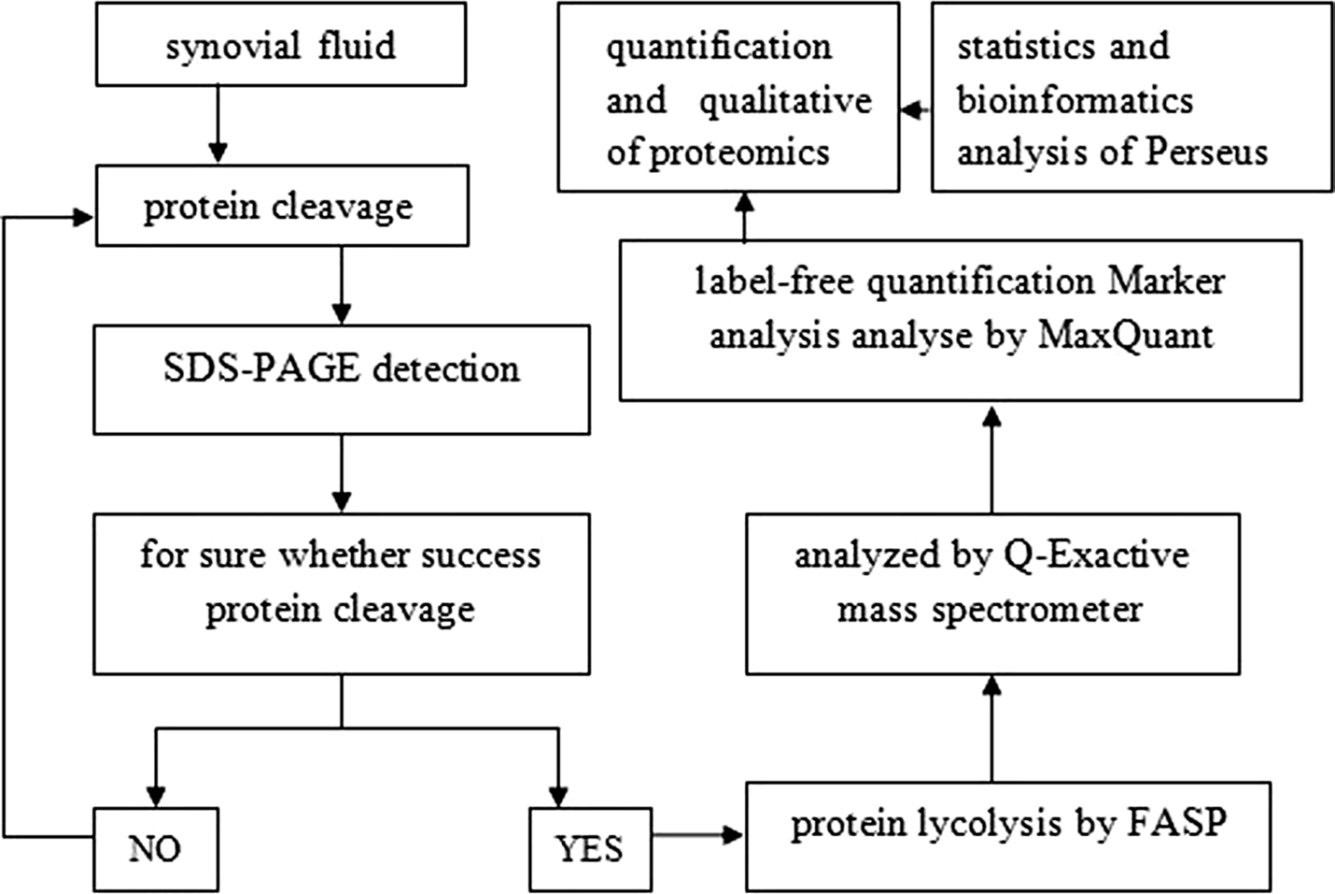
Outline of SF proteome analysis

The database files from Maxquant were analyzed by Perseus software (version 1.3.0.4). Then Gene Ontology (GO) Annotation, KEGG Pathway Annotation, Hierarchical Clustering and protein–protein interact (PPI) Network analysis were performed using the UniProtKB database (2015.03 version) and were stored in FASTA format (201504012FBOYJZXNU.fasta). We used localized sequence software, NCBI BLAST+, to compare the identification of proteins with proteins sequences in the SwissProt Mammalian Database. The whole process of SF protein detection is summarized in Fig. 1.

Quantitated protein sequences were retrieved in batches from the UniProtKB database (2015.03 version) in FASTA format (201504012FBOYJZXNU.fasta). We used localized sequence software, NCBI BLAST+, to compare the identification of proteins with proteins sequences in the SwissProt Mammalian Database. According to the similarity principle, the functional messages of homologous proteins could be used to note the target proteins. In the study, the top 10 BLAST hits with E-value less than 1e^−3^ for each query sequence were retrieved and loaded into Blast2GO (Version 2.8.0) for Gene Ontology (GO) mapping and annotation. Proteins with P-values < 0.05 and fold-change ratios ≥ 8 or ≤ 0.3 were considered as differentially expressed proteins, and were used for the following analysis.

Proteins do not independently execute their function but act in coordination with other proteins to accomplish biochemical reactions. Thus, the biological processes in cells can be systematically and wholly understood by pathway analysis. KEGG is one of the databases used to study biochemical reaction pathways. We utilized the KEGG Automatic Annotation Server to compare target protein sequences with the KEGG gene database of rabbit protein sequences and then annotated the KO numbers of homologous/similar proteins to the relevant KEGG pathways.

## Results

### The protein contents of samples and OD_280_ contents of peptides in groups A and B

The protein contents of samples in the two groups are shown in Table 2. Group A was 20.72 μg/μl, Group B was 19.67 μg/μl.

**Table 2:** The protein contents of samples and OD_280_ contents of peptides in the US and sham US groups

Group	Volume (μl)	Protein contents (μg/μl)	OD280 (μg/μl)
US group	200	20.72	1.30
Sham US group	200	19.67	1.58

### SDS-PAGE analysis

**Fig. 2:**
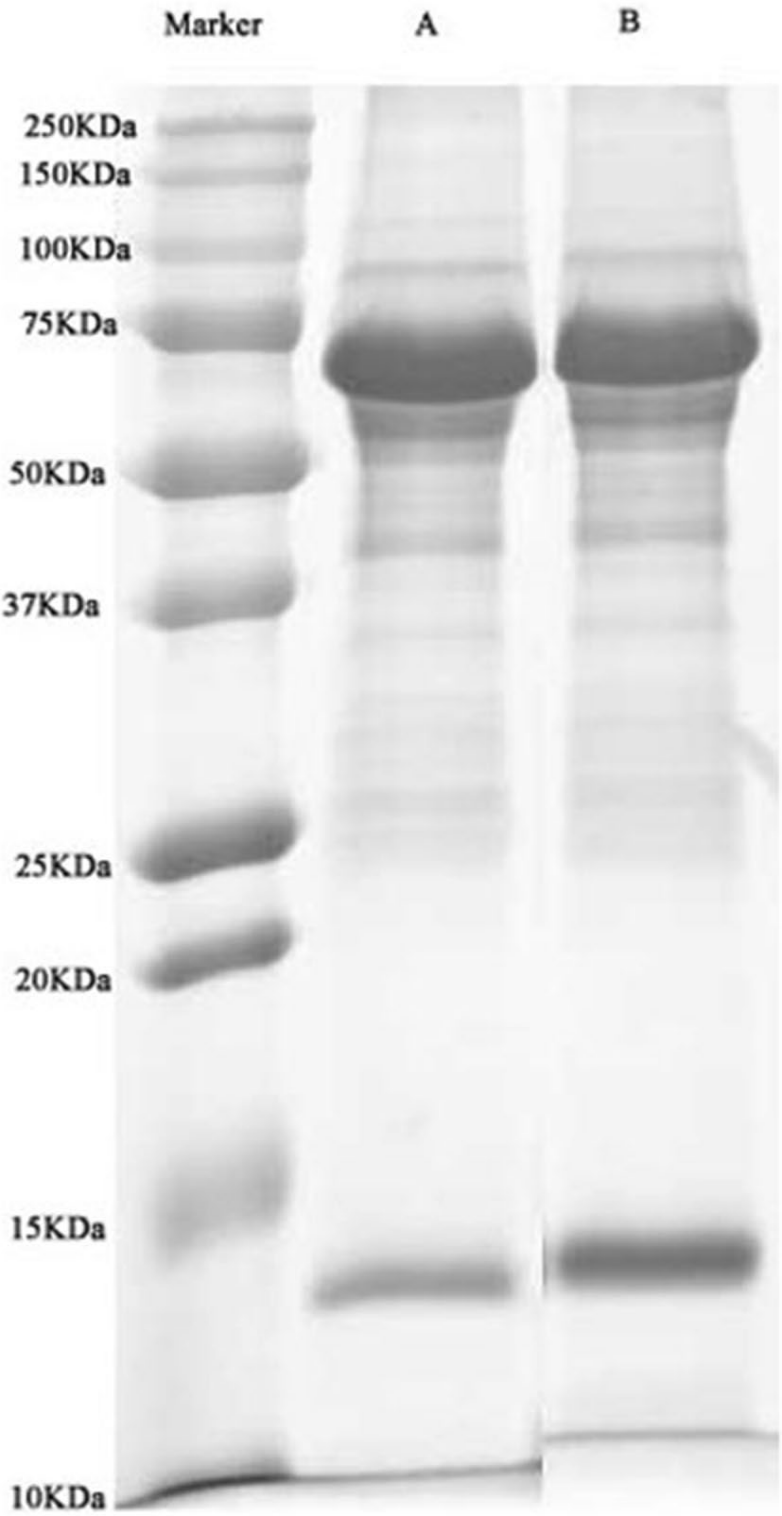
SDS-PAG electropherogram. **a** Ultrasound, **b** Control ultrasound

According to the Coomassie brilliant blue atlas, the clear separation of bands of proteins in different groups indicates well extracted protein (Fig. 2).

### Protein glycolysis analysis by LCMS/MS in groups A and B

**Fig. 3:**
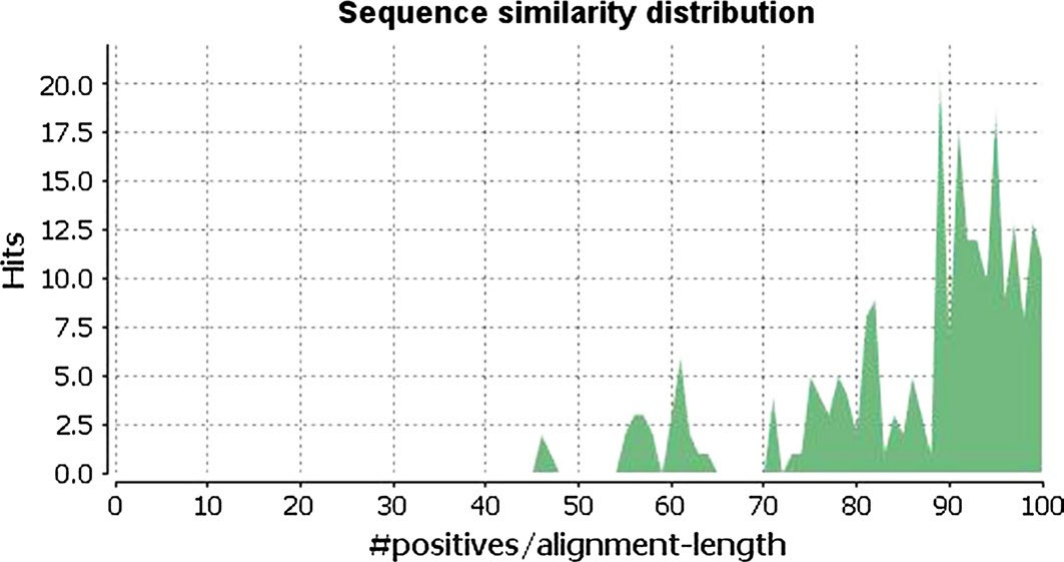
The distribution of sequences with respect to similarity

We identified 244 proteins in the two groups. We confirmed 19 significantly differentially expressed proteins between the two groups. The comparable similarity range was 47–100%; most similarity was ≥ 79% (Fig. 3).

**Fig. 4:**
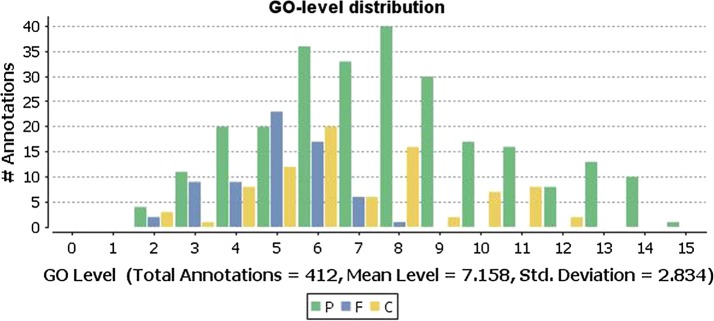
The GO level distribution of proteins. *P* process of biological, *F* function of molecular, *C* component of cellular

We extracted 769 relevant items in GO BlastGO2 for the 19 differentially expressed proteins. We annotated 18 proteins and 350 GO function items; the average level of GO was 6.731 (Fig. 4).

**Fig. 5:**
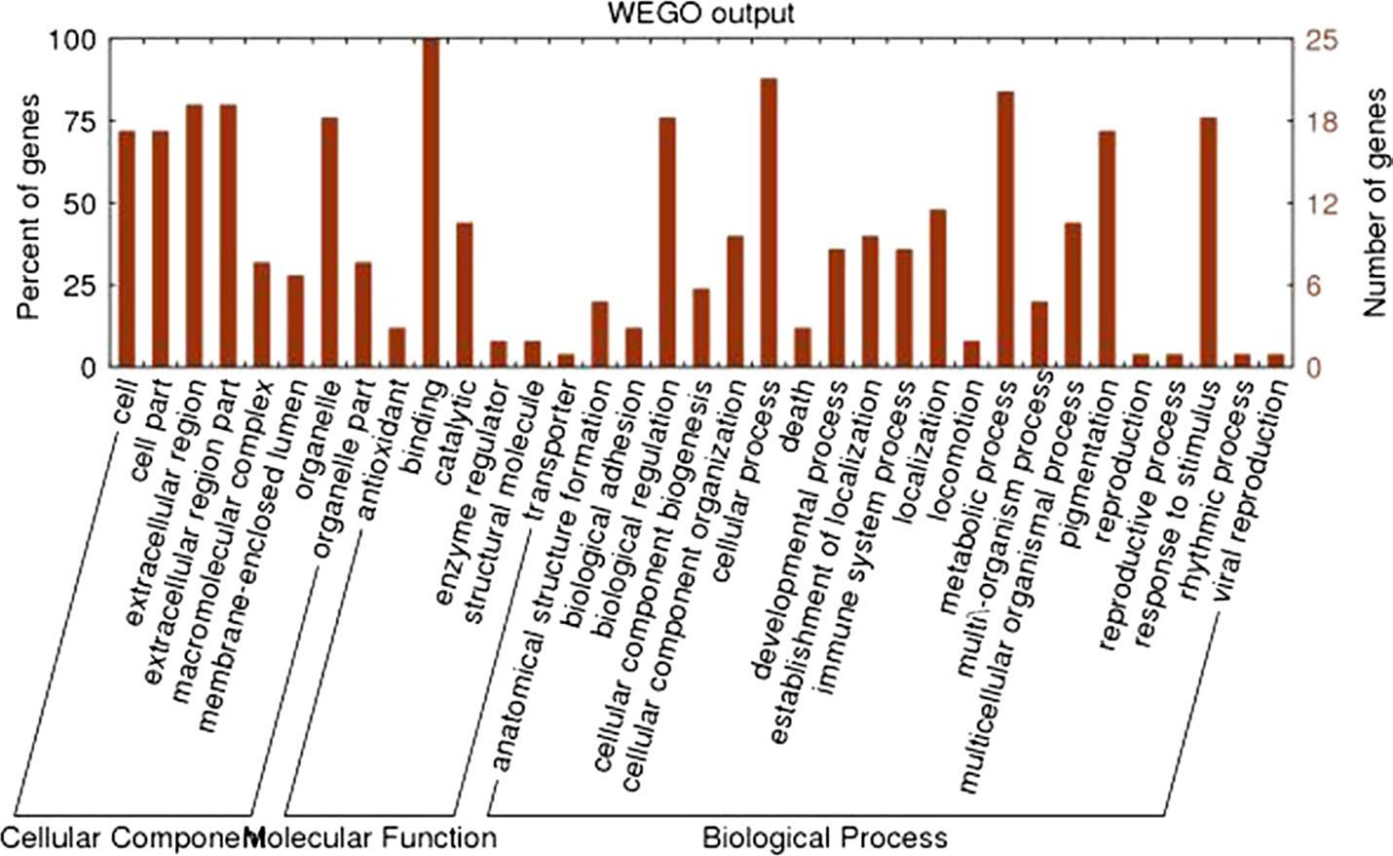
The GO level 2 protein function distribution

The final annotation resulted in 19 protein sequences annotated by 361 GO items. The GO level 2 protein function distribution is shown in Fig. 5.

We compared the target protein sequences with the KEGG database of rabbit protein sequences, then annotated the KO proteins of homologous/similar proteins to the relevant KEGG pathways. We extracted 10 significantly differentially expressed proteins sequences including 32 relevant KEGG messages/metabolism pathways (Table 3). All annotation pathways were saved as map files, and the significantly differentially expressed proteins were highlighted in green. Among the 10 proteins, the levels of six were decreased, including apolipoprotein A-I (AopA-1), transferrin (TF), carboxypeptidase B2 (CBP2), arylesterase/paraoxonase (PON), fibrinogen alpha chain, and alpha-2-macroglobulin (A2M), and the levels of four were increased including molecular chaperone HtpG/heat shock proteins (htpG, HSP90A), decorin (DCN), pyruvate kinase (PK, pyk), and fatty acid-binding protein 4/adipocyte (FABP4, aP2). The results of protein content ratios and significantly differentially expressed proteins in the US and sham US groups are shown in Table 3.

**Table 3:** Proteins content ratios of the US and sham US groups for significantly differentially expressed proteins

Protein ID	KO	Map ID	Pathway	Definition name	Ratio A/B	*P* Value
B7NZM1	K08757	ko04975 ko03320 ko05143 ko04977	Fat digestion and absorption PPAR signaling pathway African trypanosomiasis Vitamin digestion and absorption	apolipoprotein A-I	0.0618	0.000121894
G1TKE4	K14736	ko04066 ko04978	HIF-1 signaling pathway Mineral absorption	Transferring	0.1773	1.02272E−06
G1TET0	K01300	ko04610 ko04972 ko04974	Complement and coagulation cascades Pancreatic secretion Protein digestion and absorption	carboxypeptidase B2 CBP2	0.1939	0.016388579
G1SN96	K01045	ko00363 ko00627	Bisphenol degradation Aminobenzoate degradation	Arylesterase/paraoxonase	0.2037	0.00047988
G1TR31	K03910	ko04610	Complement and coagulation cascades	alpha-2-macroglobulin A2M	0.2834	0.001349259
G1T0X2	K03903	ko04610 ko04611	Complement and coagulation cascades Platelet activation	fibrinogen alpha chain FGA	0.2944	2.2664E−05
P30946	K04079	ko05215 ko04141 ko04612 ko04151 ko04626 ko04914 ko04915 ko05200 ko04621	Prostate cancer Protein processing in endoplasmic reticulum Antigen processing and presentation PI3K-Akt signaling pathway Plant–pathogen interaction Progesterone-mediated oocyte maturation Estrogen signaling pathway Pathways in cancer NOD-like receptor signaling pathway	molecular chaperone HtpG htpG, HSP90A	8.367	0.004786751
Q28888	K04660	ko04350 ko05205	TGF-beta signaling pathway Proteoglycans in cancer	Decorin DCN	11.362	0.009282328
P11974	K00873	ko00010 ko00230 ko00620 ko01200 ko01230 ko04922 ko04930	Glycolysis/Gluconeogenesis Purine metabolism Pyruvate metabolism Carbon metabolism Biosynthesis of amino acids Glucagon signaling pathway Type II diabetes mellitus	pyruvate kinase PK, pyk	12.006	0.011111008
		ko05203 ko05230	Viral carcinogenesis Central carbon metabolism in cancer			
G1T9I9	K08753	ko03320	PPAR signaling pathway	fatty acid-binding protein 4, adipocyte FABP4, aP2	15.174	7.13941E−05

## Discussion

There are many rehabilitation methods for OA, and exercise therapy, with diverse exercise protocols, plays an important role in OA rehabilitation. A number of reports have indicated that physical agents can improve the range of motion and activities of daily living [18–20]. US is an effective method for the treatment of KOA [21]. US can slow down the swelling symptoms of KOA and significantly improve joint function [22, 23]. The therapeutic effect of US for OA is superior to most other physical agents [11], and its mechanisms involve diminishing inflammatory factors, which delays cartilage matrix degradation, and inhibiting chondrocyte apoptosis, which slows cartilage degeneration [24, 25].

With the development of proteomic technologies, it is possible to reveal the mechanisms underlying therapeutic US. There are different proteomics methods to analyze tissues. The iTRAQ method can authenticate any type of protein, and the advantages of label-free quantitation methods include no need for isotope labels as the internal standard. As previously mentioned, synovial membrane lesion is a pathological factor in OA. Whether US can alter protein expression in SF has not been reported, so we investigated the effects of US on SF proteins by a label-free method. Our results indicate that US can alter protein levels in SF (Fig. 2). These proteins have different distribution and functions, as shown in Figs. 3, 4, 5, and affect the process of KOA on multiple levels and by different pathways (see Figs. 4, 5 and Table 3). Comparing the differences in protein expression in SF between US with sham US, we identified 19 functional proteins, including 10 principal proteins that participate in 32 metabolic and transduction pathways. These results demonstrate US therapy can decrease AopA-1, TF, CBP2, PON, fibrinogen alpha chain and A2M protein levels and increase HtpG/HSP90A, DCN, PK/PKY, and FABP4/aP2 protein levels in SF of KOA.

AopA-1 is sensitive to the level of TNFα, and many studies have shown that TNFα plays a very important role in OA pathology. The regulation of cholesterol efflux may be play a critical role in OA; therefore, an LXR agonist to facilitate cholesterol efflux was considered a target for OA treatment [26]. Okabe et al. [27] showed that APOL-1 mRNA was down-regulated in patients with no sign of arthritis. They considered ApoA-1 to be a vital factor in the pathogenic mechanism of OA. We identified AopA-1 as the most significantly reduced protein in SF in our KOA model. We presume that US treatment reduced AopA-1 levels (Table 3). These results are in accordance with the report of Kong [28]. AopA-1 is the most significantly down-regulated protein in osteopenic femurs [29]. AopA-1 can affect fat digestion and absorption, vitamin digestion and absorption (Table 3); all three pathways would influence the course of OA [30–32].

TF/TRF transfers iron into cells, and the correlation between TF and osteoarticular diseases has been studied. Alexander et al. [33] suggested that the role of transferrin is not vital for chondrocyte survival or matrix synthesis. Chales et al. regarded arthropathy as a major and distinctive manifestation of hemochromatosis, resembling degenerative joint disease with involvement of unusual articular sites, almost identical to the arthropathy in calcium pyrophosphate dihydrate crystals deposition disease (chondrocalcinosis); early biomarkers show increasing serum transferrin saturation [34]. Huleihel et al. found IL1 can induce TRF secretion from Sertoli cells in vitro, meaning that a reduced IL1 content would depress the TRF level [35]. In this study, we found US can depress the TF protein content in SF (Table 3); therefore, we suggest US may depress the IL level in SF, thereby reducing the TRF content, because we have previously found US can decrease levels of inflammatory factors [11]. TF affects mineral absorption and the HIF-1 signaling pathway to affect the pathological process of OA [36, 37].

The thrombin/thrombomodulin complex activates CPB. CPB acts as a procoagulant for fibrin clot formation. It can aggregate proinflammatory mediators, such as bradykinin, C5a and osteopontin. Although Song et al. [38] have shown that CPB and C5a in SF was higher in RA patients than in OA patients, CPB plays an important role in dampening local, C5a-induced inflammation and acts as a molecular link between inflammation and coagulation in OA. Christin suggested that CPB could prevent inflammation from destroying the joints in OA, by reducing complement activation to some degree [39]. In short, the relationship between CPB2 and OA has not yet been defined. We identified CBP2 in the SF of KOA and after US treatment the CBP2 protein level was depressed (Table 3). CBP2 affects tissue metabolism via complement and coagulation cascades, pancreatic secretion and protein digestion and absorption pathways [40, 41].

PONs include PON1, PON2 and PON3, and they resist the oxidation of low density lipoprotein (LDL). PON1 hydrolyzes paraoxon. Some studies have indicated decreased PON1 expression after treatment of HepG2 cells with oxydic LDL, oxydic lipidosome, IL-1β, or TNF-α [42]. PON1 activity declines under the environment of oxidative stress, which should result in chondrocyte telomere instability, thus the synthesis of functional glycosaminoglycan in chondrocytes is decreased [43]. Erturk et al. thought PON1 activation in serum may be an effective adjunctive marker of knee OA [44]. All of the above mentioned articles were about the relationship of serum PON to OA rather than to SF in OA. The results of this study indicate that US can decrease the PON protein level in SF (Table 3). We therefore speculate that after-treatment with US, the inflammatory reaction in the synovium is relieved, resulting in decreased PON secretion by inflammatory factors. PON affects metabolism by bisphenol degradation and aminobenzoate degradation pathways, but the relationships of bisphenol and aminobenzoate degradation metabolic pathways to OA have not been investigated.

A2M is a broad-spectrum proteinase inhibitor. A2M can repress the proteolytic enzyme that functions in the process of OA [45]. Most in vitro studies have verified the relationship between A2M and OA, while the direct effects of A2M on SF have not been reported in KOA in vivo. Injection of A2M into the joint cavity can prevent the destruction of articular cartilage [46]. Wang et al. [47] also found that A2M was at insufficient levels to depress the high content of catabolic factors in OA SF; however, complementary intraarticular A2M offers chondral protection in post-traumatic OA. However, our results indicate US can decrease the A2M protein level in SF (Table 3). We speculate that after US treatment, synovial inflammation is reduced and the levels of anti-inflammatory cytokines are decreased by a feedback mechanism. For example, Kolarov et al. [48] demonstrated an increase in A2M in OA blood and SF as a protective reaction. A2M also affects tissue metabolism by complement and coagulation cascade pathways.

Fibrinogen is involved in hemokinesis and Kong have identified three proteins (apolipoprotein, haptoglobin precursor, and fibrinogen D fragment) that are related to joint diseases in OA SF samples by one and two dimension electrophoresis [18]. Belcaro et al. found fibrinogen levels were lowered to 62.8% of initial values after intervention in response to the anti-inflammatory substance, pycnogenol, in osteoarthritic joints [49]. Balakrishnan found that fibrinogen content was increased in rheumatic arthritis (RA) SF but not in OA SF [7]. Richette reported that fibrinogen was decreased after massive loss weight in obese patients who suffer from knee OA [50]. Our results indicate US can decrease the fibrinogen protein level in SF (Table 3). We speculate US can decrease fibrinogen levels to relieve synovium membrane inflammation. Fibrinogen affects tissue metabolism by complement and coagulation cascades and platelet activation pathways.

There are many kinds of heat shock protein according to molecular weight and homology and they include HSP90, HSP70 and HtpG/HSP90A. They act to prevent harm to organisms [51]. Apaf-1 and TNF are closely related to OA. HSP90 blocks apoptosis of cells by repressing the oligomerization of Apaf-1 [52]. HSP90 can also prevent the apoptosis induced by TNF by maintaining the receptor protein stability and interaction to enhance NF-kB activity and regulate AKT [53]. Therefore, HSPs affect the differentiation, maturation and metabolism of chondrocytes. The expression of HSP70 and aggrecan were increased in model OA rats administrated microwave radiation and intraarticular glutamine [54]. No relationship between SF and OA and HSPs was previously reported, but our results indicate that SF contains HSPs/90 and that the HSPs/90 content was increased in the US group compared with the sham US group (Table 3) so that synovial inflammation is relieved. HSPs/90 influences tissue metabolism by processing proteins involved in antigen processing and presentation, endoplasmic reticulum function, NOD-like receptor signaling, PI3K-Akt signaling, plant–pathogen interaction, progesterone-mediated oocyte maturation and estrogen signaling.

DCN is a family of small leucine rich proteoglycans; most DCNs are distributed in bone, cartilage, tendon and sclera in the extracellular matrix [55]. DCN has a feedback role on TGF-β function which affects the synovial membrane and cartilage [56]. DCN mRNA content increases and DCN protein levels decrease in the cartilage of OA patients and with increasing age, the decomposition of DCN fragments increases [57]. These results indicate that DCN plays an important role in cartilage repair in OA patients. Our results show that the DCN protein is enhanced in SF (Table 3); therefore, US can prevent synovial damage. DCN influences metabolism via the TGF-beta signaling pathway.

PK/PYK is a key enzyme in glycolysis. There are four PK isoenzymes, LPK, RPK, M1PK and M2PK [58]. Recently, Bluemlein revealed that M2PYK is nearly always the most plentiful subtype. PKM2 catalyzes the conversion of phosphoenolpyruvate to pyruvate in the glycolysis pathway [59]. PKM2 regulates cell proliferation [60]. Oremek et al. [61] found the pyruvate kinase concentrations in plasma were increased in patients with rheumatic diseases, but the relationship between pyruvate kinase and OA hasn’t been reported. Our results state that US can stimulate PYK protein in SF (Table 3), which would prevent synovial damage. PKY influences metabolism via glycolysis, purine metabolism, carbon metabolism, biosynthesis of amino acids, and diabetes mellitus pathways.

FABP4/aP2 belongs to a family of low-molecular-weight cytoplasmic proteins and mainly affects the PPAY-γ pathway. Many diseases are intimately related to FABP4/aP2 (e.g. metabolic syndrome and atherosclerosis and possibly osteoarthritis and FABP) [62, 63], but its involvement in SF in KOA is unknown. Our investigation found US can upregulate FABP4/aP2 protein in SF (Table 3).

## Conclusion

In conclusion, our results demonstrate that US can downregulate ApoA-I, TF, CBP2, PON, A2M, and fibrinogen alpha chain levels, upregulate HtpG/HSP90A, DCN, PK/PKY, FABP4/aP2 protein levels in SF of KOA, those proteins may serve as targets to inhibit the progress of KOA. But further investigation is needed to identify the most important metabolic pathways in SF and how US treatment of KOA affects these pathways.

## Abbreviations

US: ultrasound
OA: osteoarthritis
KOA: knee osteoarthritis
SF: synovial fluid
ACLT: anterior cruciate ligament transaction
MS: mass spectrometry
iTRAQ: isobaric tags for relative and absolute quantification
GO: Gene Ontology
KEGG: Kyoto Encyclopedia of Genes and Genomes
AopA-1: apolipoprotein A-I
TF: transferring
CBP2: carboxypeptidase B2
PON: arylesterase/paraoxonase
A2M: alpha-2-macroglobulin
HSP: heat shock proteins
DCN: decorin
PK: pyk, pyruvate kinase
